# In-Hospital Mortality Among 40,253 Older Adults with Hip Fracture: Survival Outcomes and Multivariate Analysis in a Chilean Cohort

**DOI:** 10.3390/jcm14217717

**Published:** 2025-10-30

**Authors:** Eduardo Guzmán-Muñoz, Yeny Concha-Cisternas, Manuel Vásquez-Muñoz, Rodrigo Yañez-Sepúlveda, Cristian Núñez-Espinosa, Sacha Bittelman Saporte, Fernando Nemtala Urquiza, Rodrigo Morales Araneda

**Affiliations:** 1 Escuela de Kinesiología, Facultad de Salud, Universidad Santo Tomás, Talca 3460000, Chile; eguzmanm@santotomas.cl; 2 Escuela de Pedagogía en Educación Física, Facultad de Educación, Universidad Autónoma de Chile, Talca 3460000, Chile; 3 Vicerrectoría de Investigación e Innovación, Universidad Arturo Prat, Iquique 1100000, Chile; 4 Center for Health Data Observation and Analysis (CADS), School of Medicine and Health Sciences, Universidad Mayor, Santiago 8580745, Chile; 5 Escuela de Medicina, Facultad de Medicina y Ciencias de la Salud, Universidad Mayor, Santiago 8580745, Chile; sacha.bittelman@umayor.cl; 6 Facultad de Educación y Ciencias Sociales, Universidad Andrés Bello, Viña del Mar 2200055, Chile; rodrigo.yanez.s@unab.cl; 7 School of Medicine, Universidad Espíritu Santo, Samborondón 092301, Ecuador; 8 Escuela de Medicina, Universidad de Magallanes (UMAG), Punta Arenas 6200000, Chile; cristian.nunez@umag.cl; 9 Centro Asistencial Docente e Investigación, Universidad de Magallanes, Punta Arenas 6200000, Chile; 10 Clínica Red Salud Vitacura, Santiago 8580745, Chile; fnemtala@yahoo.com (F.N.U.); rodrigo.morales.a@gmail.com (R.M.A.)

**Keywords:** hip fractures, aged, comorbidity, hospitalization, mortality, health care systems

## Abstract

**Background:** Hip fracture is a common geriatric condition associated with disability, institutionalization, and mortality. In-hospital mortality reflects both patient vulnerability and the quality of care, yet evidence from Latin America is scarce. **Objective:** We aimed to identify factors associated with in-hospital mortality in Chilean older adults with hip fractures. **Methods:** We conducted a retrospective cohort study using the Chilean National Health Fund (FONASA) database, which included patients aged 60 years or older who were hospitalized with a hip fracture (ICD-10 S72.0–S72.2) between 2019 and 2024. Variables analyzed included age, sex, surgical treatment, number of comorbidities, Diagnosis-Related Group (DRG) severity level, and relative weight. Survival was evaluated with Kaplan–Meier curves and log-rank tests. Multivariable Cox proportional hazards models estimated adjusted hazard ratios (HR) with 95% confidence intervals (CI). **Results:** The cohort comprised 40,253 patients (76.8% women; mean age 81.9 ± 9.1 years). Overall, in-hospital mortality was 3.5%. Independent predictors of mortality included absence of surgery (HR = 9.56; 95% CI: 8.38–10.90), higher DRG severity level (HR = 3.87; 95% CI: 3.42–4.37), advanced age (HR per year = 1.05; 95% CI: 1.04–1.05), male sex (HR = 1.12; 95% CI: 1.03–1.27), and multimorbidity (≥3 comorbidities; HR = 2.73; 95% CI: 1.98–3.99). **Conclusions:** Timely surgery and stratification with administrative indicators (DRG) are key to reducing in-hospital mortality. The findings support strengthening orthogeriatric models in Chile.

## 1. Introduction

Falls represent one of the most frequent geriatric events and have a major health impact in older adults, as they are associated with severe injuries, functional impairment, and an increased risk of institutionalization [1,2]. Among their consequences, hip fracture is recognized as one of the main markers of geriatric vulnerability due to its significant impact on health and healthcare systems [3,4]. Beyond the bone injury itself, this event often triggers a rapid loss of autonomy, the onset of long-term functional dependence, and a higher likelihood of institutionalization in long-term care facilities, all of which result in a substantial increase in healthcare and social costs [5,6]. Nevertheless, its implications extend beyond functional and economic spheres, as it also constitutes a clinical event frequently accompanied by severe complications and a high in-hospital mortality [7].

In-hospital mortality following hip fracture is a multifactorial phenomenon, determined by the interaction between patient clinical characteristics, comorbidity burden, and the quality of immediate care processes [7,8]. This outcome has been recognized as critical, as it reflects both the clinical vulnerability of older adults and the efficiency of the hospital system [9].

In large international cohorts, it has been estimated that between 2% and 4% of patients admitted with hip fracture die during hospitalization, with variations attributable to comorbidity burden, prior functional status, and the occurrence of acute medical complications [7,8]. Similarly, a recent analysis reported that, despite a reduction in 30-day mortality over the past decades, in-hospital mortality among individuals with hip fracture has remained stable at approximately 3%, being higher in men and in patients with greater comorbidity burden [8]. Complementarily, research has identified immediate clinical factors at admission—such as surgical treatment delay, preoperative anemia, and impaired renal function—as independent predictors of in-hospital death. More recently, a multicenter study conducted in Italy demonstrated that the presence of cardiovascular comorbidities, renal impairment, and respiratory complications was a key determinant of increased in-hospital mortality [10].

Considering this evidence, countries in Europe, Asia, and North America have developed integrated orthogeriatric care models aimed at reducing both short- and long-term complications and mortality [11]. However, in Latin America, the information available to guide the development of such strategies remains limited. In Brazil, in-hospital mortality rates of approximately 14% and up to 23% at one year have been documented, mainly associated with advanced age, comorbidities, and in-hospital complications [12], In Chile, healthcare is organized through a mixed public–private system, where the National Health Fund (FONASA) covers approximately 85% of adults aged ≥60 years. Most hip fracture cases are treated in public hospitals, where challenges such as surgical delays, limited orthogeriatric coordination, and unequal access to post-operative rehabilitation persist. These characteristics of the Chilean healthcare system may influence clinical outcomes and justify the need for national evidence on in-hospital mortality among older adults with hip fracture. Based on these considerations, the objective of this study was to determine the factors associated with in-hospital mortality due to hip fracture in a cohort of Chilean older adults. We hypothesized that clinical and healthcare-related factors previously reported in the literature—such as older age, comorbidity burden, higher diagnostic severity, and absence of surgical treatment—would be associated with an increased risk of in-hospital mortality among Chilean older adults with hip fracture.

## 2. Materials and Methods

This retrospective cohort study analyzed data from the public database of the Chilean National Health Fund (FONASA), which compiles anonymized hospital discharge records from all public hospitals in Chile.

This study included data from 72 public hospitals across Chile, all of which belong to the national healthcare network administered by the National Health Fund (FONASA). These hospitals provide care for moderate- to high-complexity cases, including trauma and orthopedic surgery, and have access to multidisciplinary teams for managing hip fractures in older adults. All hospitalizations of adults aged 60 years and older with a principal diagnosis of hip fracture recorded in the FONASA database between 1 January 2019, and 31 December 2024, were included. Records were excluded if they contained missing information on age, sex, or discharge status, if the diagnosis was outside the specified codes, or if they represented transfers or duplicate admissions. Data validation procedures included cross-checking unique admission and discharge identifiers to ensure internal consistency, prevent duplication, and confirm the accuracy of hospitalization records.

The analysis was limited to hospitalizations registered in the public healthcare system (FONASA), which covers approximately 85% of Chile’s population aged ≥60 years. Patients treated in private hospitals (ISAPRE system) were not included, as private healthcare providers do not share clinical or administrative data with the national health information system. Consequently, the findings primarily reflect outcomes from the public healthcare sector, where the majority of hip fractures among older adults occur.

### 2.1. Data Collection

Hospital discharge records were obtained from the publicly available database of the FONASA (https://datosabiertos.fonasa.cl, accessed on 8 August 2025), which compiles anonymized information from all public hospitals in the country. Patients aged 60 years or older with a principal diagnosis of hip fracture were identified using the International Classification of Diseases, 10th Revision (ICD-10) codes S72.0 (fracture of the neck of the femur), S72.1 (pertrochanteric fracture), and S72.2 (subtrochanteric fracture). Diagnostic and procedural codes in the FONASA database follow the ICD-10. Data entry and coding are routinely monitored by the Chilean Ministry of Health, which conducts periodic audits and quality checks to ensure internal consistency and accuracy across hospital records. These validation procedures support the reliability of the administrative data used in this study.

**Figure 1:**
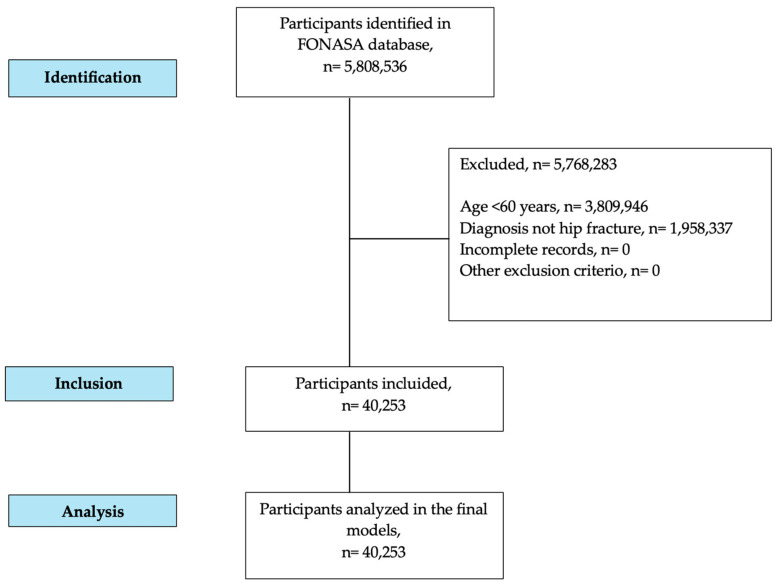
STROBE flowchart of participants.

The FONASA database includes standardized fields containing demographic, clinical, and administrative information, as well as hospital outcome (discharged alive or deceased) (Figure 1). All variables analyzed in this study were extracted directly from these standardized records, eliminating the need for chart review or additional validation by imaging reports. Mortality data correspond exclusively to in-hospital deaths recorded at the time of discharge; no post-discharge follow-up was performed.

### 2.2. Variables

#### 2.2.1. Dependent Variable

The dependent variable was in-hospital mortality, recorded in the FONASA hospital discharge database as the discharge condition (0 = survived, 1 = deceased). Time-to-event was defined as the length of hospital stay in days, calculated from the date of admission to the date of discharge or death. Mortality data correspond exclusively to deaths occurring during hospitalization; no post-discharge follow-up was performed.

#### 2.2.2. Independent Variables

The independent variables extracted from the FONASA database were as follows: (i) Sociodemographic variables: Sex (male or female) and age (analyzed as a continuous variable and categorized into age groups 60–69, 70–79, 80–89, and ≥90 years); (ii) Clinical and administrative variables: (a) Surgical treatment: categorized as yes (surgery performed) or no (no surgical intervention recorded); (b) Number of comorbidities: calculated from the count of secondary diagnoses recorded at discharge and categorized into <3 or ≥3 comorbidities [12]; (c) Severity level: obtained from the Chilean Diagnosis-Related Groups (DRG) classification, ranging from mild to severe, based on expected resource use and clinical complexity; (d) Relative weight: a unitless index from the DGR system representing the expected resource consumption for each hospitalization relative to the national average.

The comorbidities variable was operationalized as the total count of secondary diagnoses recorded at discharge, providing a general estimate of the patient’s disease burden based on administrative data. Although this differs from standardized comorbidity indices such as the Charlson or Elixhauser, which assign weighted scores according to disease severity, this approach allows consistent quantification across all records in the FONASA dataset, where detailed clinical parameters are unavailable.

### 2.3. Statistical Analysis

Statistical analyses were performed using GraphPad Prism, version 9 (GraphPad Software, San Diego, CA, USA). The analysis of in-hospital mortality was conducted in two stages: descriptive and analytical. In the descriptive stage, continuous variables were expressed as mean and standard deviation, while categorical variables were summarized as absolute and relative frequencies. For the analytical stage, time-to-event analysis was performed using the Kaplan–Meier method to estimate survival functions for the main variables of interest. Differences between survival curves were assessed using the log-rank (Mantel–Cox) test, and the Gehan–Breslow–Wilcoxon test was additionally reported to provide greater sensitivity to early events. Median survival times were calculated when applicable. Cox proportional hazards regression models were used to estimate unadjusted and adjusted hazard ratios (HR) with their corresponding 95% confidence intervals (CI). Variables with *p* < 0.20 in univariate Cox models were entered into the multivariate models. The proportional hazards assumption was verified for all variables included in the final model. This assumption was evaluated using Schoenfeld residuals and visual inspection of log–log survival plots for key variables, including surgical treatment and DRG severity level. No relevant deviations from proportionality were observed, supporting the adequacy of the Cox model. Kaplan–Meier survival curves were generated for variables that remained statistically significant in the adjusted Cox model. For all analyses, a *p*-value < 0.05 was considered statistically significant.

## 3. Results

Table 1 summarizes the baseline characteristics of the study population. The sample consisted of 40,253 patients with femoral fractures, of whom 76.8% were women. The overall in-hospital mortality was 3.5%. When stratified by sex, mortality was higher among men (4.4%) than among women (3.2%). Additional baseline information stratified by hospital outcome and surgical treatment is presented in Supplementary Tables S1 and S2.

**Figure 2:**
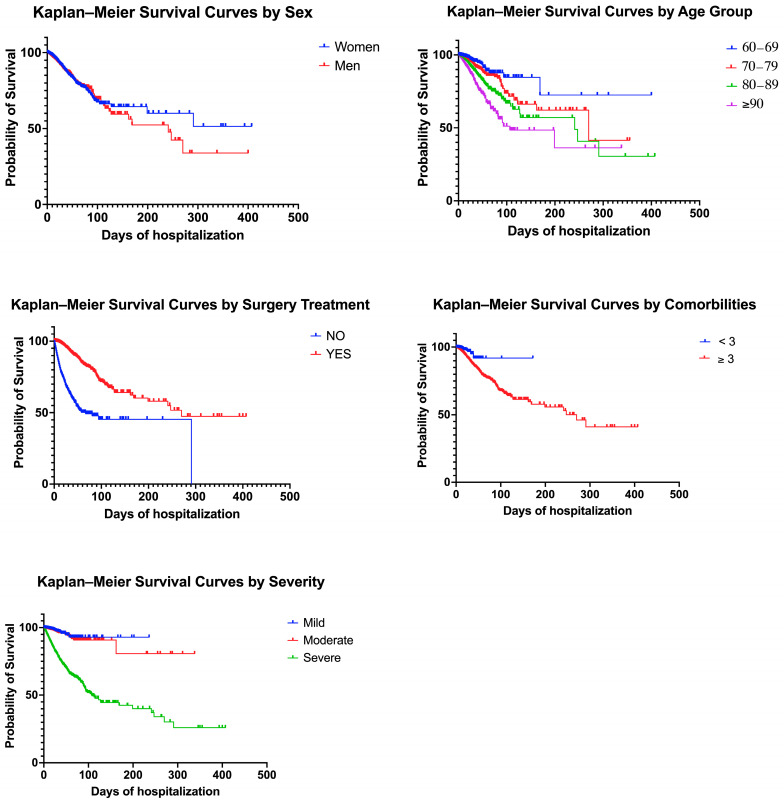
Kaplan–Meier survival curves for in-hospital mortality after hip fracture in older adults. Time represents the number of hospitalization days from admission to discharge or death.

Figure 2 shows the Kaplan–Meier survival curves for the main variables analyzed, and Table 2 summarizes the results of the Cox proportional hazards models, both unadjusted and adjusted. Regarding sex, the curves showed a higher survival probability for women compared with men, with a statistically significant difference (log-rank χ^2^ = 5.94; *p* = 0.0148; Gehan–Breslow–Wilcoxon χ^2^ = 13.83; *p* = 0.0002). When evaluating early mortality, in-hospital mortality rates at 7, 14, and 30 days were consistently higher in men than in women (1.8% vs. 1.2% at 7 days, 2.8% vs. 2.1% at 14 days, and 3.7% vs. 2.8% at 30 days, respectively). Median survival was undefined for women and men. In the adjusted Cox model, male sex was associated with a 12% higher risk of in-hospital mortality (HR = 1.12; 95% CI: 1.03–1.27; *p* < 0.001). For age, a progressive decrease in survival was observed with increasing age group, with significant differences (log-rank χ^2^ = 363.7; *p* < 0.0001; trend test χ^2^ = 326.8; *p* < 0.0001). The adjusted model revealed that each additional year of age was associated with a 5% increase in the risk of in-hospital mortality (HR per year = 1.05; 95% CI: 1.04–1.05; *p* < 0.001).

Regarding the surgical treatment, the Kaplan–Meier curves showed markedly lower survival in patients who did not undergo surgery (log-rank χ^2^ = 2914; *p* < 0.0001; Gehan–Breslow–Wilcoxon χ^2^ = 3828; *p* < 0.0001), with a median survival of 67 days compared with 270 days in those who underwent surgical intervention. The unadjusted analysis showed an HR of 10.91 (95% CI: 9.81–12.12; *p* < 0.001), and in the adjusted model, absence of surgical treatment was associated with a 9.56-fold higher risk of in-hospital mortality (95% CI: 8.38–10.90; *p* < 0.001). Similarly, the number of comorbidities had a significant impact: patients with ≥3 comorbidities had lower survival than those with fewer than three (log-rank χ^2^ = 46.10; *p* < 0.0001), with an adjusted HR of 2.73 (95% CI: 1.98–3.99; *p* < 0.001). Severity level according to DRG was also strongly associated with mortality, with a significant reduction in survival as severity increased (log-rank χ^2^ = 1424; *p* < 0.0001; trend test χ^2^ = 1106; *p* < 0.0001). In the adjusted model, each one-level increase in DRG severity was associated with a 3.87-fold higher risk of in-hospital mortality (95% CI: 3.42–4.37; *p* < 0.001). Finally, relative weight was also significant in the adjusted model, where each additional unit of relative weight was associated with a 15% higher risk of in-hospital mortality (HR = 1.15; 95% CI: 1.09–1.22; *p* < 0.001).

## 4. Discussion

In this large national cohort of 40,253 patients aged 60 years and older hospitalized with hip fractures in 72 public hospitals across Chile, the overall in-hospital mortality rate was 3.5%. Survival analysis showed that male sex, older age, absence of surgical treatment, having three or more comorbidities, higher DRG severity level, and higher relative weight were independently associated with an increased risk of in-hospital death. In the adjusted Cox model, absence of surgical treatment and higher DRG severity level emerged as the strongest predictors, with hazard ratios of 9.56 and 3.87, respectively. Age demonstrated a clear dose–response relationship, with each additional year increasing the mortality risk by 5%. In comparison, male sex and having three or more comorbidities were associated with a 12% and 173% higher risk of death, respectively. Each additional unit of relative weight increased the risk of mortality by 15%. These findings highlight the multifactorial nature of in-hospital mortality following hip fracture and underscore the relevance of both clinical and administrative variables for risk stratification and management in this patient population.

In this study, in-hospital mortality due to hip fracture was 3.5%, a value that falls within the 2–4% range reported in large international cohorts [7,8,13]. However, there are reports of mortality rates varying between 6.2% and 13.3% [14,15,16,17,18]. Studies from other regions have documented even higher figures, reaching 37.2% among hospitalized older adults with hip fracture in Turkey [19] and 29% in Poland [20]. This illustrates the significant variability in outcomes across different healthcare systems. In Latin America, although mortality rates are generally lower than those reported in some European and Asian cohorts, heterogeneous values have also been observed, ranging from approximately 14% in Brazil [12], to between 1.2% and 6.5% in Argentina [21]. In the regional context, the comparatively low in-hospital mortality reported in our study is an encouraging indicator of effective early management and hospital care within the Chilean public system. This position, intermediate between the rates observed in Brazil and Argentina, likely reflects the coexistence of extensive public coverage with residual heterogeneity in hospital resources and access to timely surgery and rehabilitation. These findings reinforce the need to consolidate orthogeriatric care pathways and to design context-specific policies aimed at improving perioperative management across the region.

Another relevant finding was that the absence of surgery was associated with a 9.56-fold increase in in-hospital mortality and with a reduction in median survival to 67 days, compared with 270 days in surgically treated patients. In the United States, Chlebeck et al. (2019) reported that patients who did not undergo surgery had an in-hospital mortality rate of 15.8%, compared with 4.4% among those treated surgically [22]. Concordant results were observed in an analysis conducted in Thailand, including more than 120,000 older patients hospitalized for hip fracture, where in-hospital mortality was 2.1% in non-operated patients compared with 1.8% in those who underwent surgery [7].

These results highlight surgery as a protective strategy against mortality [7]. Not undergoing surgical intervention often implies a greater inability to mobilize, which favors complications such as deep vein thrombosis, pressure ulcers, pneumonia, or urinary tract infections, all of which are associated with an increased risk of in-hospital death [23,24]. Likewise, patients who are not operated on typically present with more severe clinical conditions or multiple comorbidities that contraindicate surgery, thereby increasing their vulnerability and explaining the higher mortality rates observed in this group [7]. However, this association should be interpreted with caution, as the absence of surgery likely reflects underlying frailty or medical contraindications rather than a modifiable determinant. In many cases, non-surgical management is selected for individuals with severe comorbidities, hemodynamic instability, or limited functional reserve, all of which inherently increase mortality risk. Thus, the absence of surgical treatment should be viewed as a proxy indicator of clinical vulnerability rather than a causal factor influencing in-hospital mortality.

On the other hand, age showed a dose–response relationship with in-hospital mortality. This finding is consistent with Guiloff et al. (2023), who reported a 5% increase in mortality risk (HR = 1.05; 95% CI: 1.04–1.06; *p* = 0.01) for each additional year of life [25], while Wu et al. (2024) found that 30-day mortality progressively increased across older age groups, even after adjusting for comorbidities and socioeconomic factors [8]. Clinically, this effect can be attributed to reduced physiological reserve, age-related sarcopenia, diminished immune responsiveness, and a greater burden of chronic diseases—all of which increase vulnerability to hospital complications [22,26,27].

Complementarily, male sex was associated with a 12% higher risk of mortality, in line with international studies. Studies from Asia and Europe have similarly reported increased mortality risk among male patients [7,8,28]. These differences in mortality prevalence by sex may be attributed to men having a higher prevalence of cardiovascular comorbidities, greater clinically unrecognized frailty, disparities in access to or adherence with postoperative care, as well as pulmonary diseases, cancer, and other conditions [25,29,30]. From a public health standpoint, male sex should be considered a high-risk marker for adverse outcomes, supporting targeted strategies for early monitoring, rehabilitation, and complication prevention in this subgroup.

Instead, multimorbidity, defined as the presence of three or more chronic diseases, was associated with an almost threefold higher risk of in-hospital mortality. This finding is consistent with recent reports indicating that a higher comorbidity index constitutes one of the strongest predictors of adverse outcomes after hip fracture [8,12,22,31]. From a clinical perspective, the accumulation of chronic conditions increases patient frailty. It predisposes to complications both during hospitalization and in the subsequent follow-up, including pneumonia, urinary tract infections, sepsis, and surgical site infections [32]. These complications, which may manifest early during hospitalization and extend up to one year after the fracture, play a decisive role in increasing mortality risk [33]. In this context, pre-existing conditions such as heart failure, coronary artery disease, dementia, chronic kidney disease, and cancer have been shown to be associated with poorer prognosis and increased mortality even up to two years after the event [22].

Finally, and consistent with the literature employing administrative hospitalization classifications, we observed that a higher DRG severity level was independently associated with nearly a fourfold increase in in-hospital mortality (HR = 3.87; 95% CI: 3.42–4.37; *p* < 0.001). In hip fracture cohorts, systems such as the All Patient Refined Diagnosis-Related Groups (APR-DRG), conceptually analogous to the DRG severity level, capture the patient’s clinical severity, are associated with worse outcomes (e.g., longer hospital stay), and are also used for in-hospital mortality adjustment [34]. Therefore, integrating these administrative metrics with classical clinical indicators enhances risk stratification and may guide more intensive interventions in highly complex subgroups.

From a clinical and policy perspective, our findings underscore the need to strengthen early risk stratification and integrated management of older adults hospitalized with hip fracture. Variables such as age, comorbidity burden, and absence of surgical treatment—identified as independent predictors of in-hospital mortality—could be incorporated into simple screening tools at admission to prioritize surgical timing, multidisciplinary assessment, and postoperative monitoring. At the system level, implementing orthogeriatric care models and monitoring in-hospital mortality as a quality indicator could help optimize resource allocation and improve outcomes within the Chilean public healthcare network.

In Chile’s public system, our results justify scaling early risk stratification at admission and orthogeriatric co-management to prioritize timely surgery, prevent complications, and standardize discharge to rehabilitation. These recommendations are consistent with national policies and local evaluations of multidisciplinary pathways reporting improved perioperative processes and outcomes.

### Strengths and Limitations

This study has several strengths. First, it analyzed a large national cohort comprising more than 40,000 patients hospitalized for hip fracture in 72 public hospitals across Chile, which provides representativeness and relevance for the healthcare system. Second, the outcome assessed—in-hospital mortality—corresponds to a standardized clinical event that is mandatorily recorded and internationally comparable. Third, the results were consistent with previous international reports, supporting the external validity of the findings and reinforcing their applicability in other contexts.

However, certain limitations must be acknowledged. The administrative nature of the database prevented access to key clinical information, such as socioeconomic status, time to surgery, baseline functional and cognitive status, or specific complications that occurred during hospitalization, which introduces a risk of residual confounding. In addition, potential misclassification of administrative variables cannot be ruled out. Diagnostic and procedural coding in large administrative datasets may be subject to recording or classification errors, which could lead to underreporting or overestimation of certain conditions. Nevertheless, these limitations are unlikely to affect the main findings, as the FONASA dataset undergoes periodic audits for consistency and completeness.

Multimorbidity was defined solely through the count of diagnoses. Although this approach is frequently used in the literature, it does not account for the relative severity or prognostic weight of each condition, which may have reduced the precision of risk estimates and limited comparability with other studies; in this regard, future research should consider the use of validated indices, such as Charlson or Elixhauser. Another potential source of bias is that mortality was assessed only during hospitalization. Deaths occurring after discharge were not captured in the database, which may lead to underestimation of total short-term mortality. This limitation is particularly relevant if early discharge is influenced by clinical stability or healthcare system factors. Nonetheless, in-hospital mortality remains a robust indicator of acute care quality and patient vulnerability, and it is the only mortality endpoint systematically available in national administrative records.

As the analysis was restricted to hospitalizations from the public healthcare system, generalization to patients treated in private hospitals should be made with caution, although the public sector concentrates the majority of hip fracture cases in Chile.

Future research should integrate administrative and clinical registries to enhance the granularity of available data. The inclusion of standardized measures of functional, cognitive, and nutritional status—along with perioperative variables such as time to surgery and postoperative complications—would allow for more accurate risk adjustment and a deeper understanding of mortality determinants in older adults with hip fracture.

## 5. Conclusions

In this national cohort of older adults with hip fractures, in-hospital mortality was 3.5%, placing it within favorable international ranges. Absence of surgery and higher DRG levels were the strongest predictors of death, along with multimorbidity, advanced age, and male sex. These findings underscore the importance of ensuring timely access to surgery, implementing comprehensive geriatric assessments upon admission, and considering the use of administrative indicators, such as DRG severity level, to enhance risk stratification. Taken together, this evidence provides key information to guide public policies, optimize clinical protocols, and strengthen orthogeriatric care models aimed at reducing in-hospital mortality among older adults with hip fractures in Chile.

## Figures and Tables

**Table 1:** Baseline characteristics of the study population.

Variable	n = 40,253
Age (years) [mean (DS)]	81.94 (9.08)
Age Group	
60–69 [n (%)]	4502 (11.18%)
70–79 [n (%)]	10,124 (25.15%)
80–89 [n (%)]	16,825 (41.80%)
≥90 [n (%)]	8802 (21.87%)
Sex	
Women [n (%)]	30,908 (76.78%)
Men [n (%)]	9345 (23.22%)
Hospital outcome	
Survived [n (%)]	38,840 (96.49%)
Deceased [n (%)]	1413 (3.51%)
Surgical treatment	
Yes [n (%)]	35,440 (88.04%)
No [n (%)]	4813 (11.96%)
Comorbidities	6.59 (4.24)
<3 [n (%)]	4215 (10.47%)
≥3 [n (%)]	36,038 (89.53%)
Severity level-DRG	
Mild [n (%)]	15,985 (39.71%)
Moderate [n (%)]	16,150 (40,12%)
Severe [n (%)]	8038 (19.97%)
Unclassified [n (%)]	80 (0.20%)
Length of hospital stay (days) [mean (DS)]	12.73 (13.94)
Relative weight (Unitless) [mean (DS)]	0.54 (0.01)

n: number; %: percentage; DS: standard deviation; DRG: Diagnosis-Related Group.

**Table 2:** Unadjusted and adjusted hazard ratios for in-hospital mortality from Cox proportional hazards models.

Variable	HR (Unadjusted)	*p*	HR (Adjusted)	*p*
Age (per year)	1.07 (1.06–1.07)	<0.001	1.05 (1.04–1.05)	<0.001
Sex (male)	1.17 (1.04–1.31)	0.009	1.12 (1.03–1.27)	<0.001
Surgical treatment (no)	10.91 (9.81–12.12)	<0.001	9.56 (8.38–10.90)	<0.001
Comorbidities (≥3)	3.27 (2.33–4.77)	<0.001	2.73 (1.98–3.99)	<0.001
Severity level-DRG (per level)	4.66 (4.21–5.15)	<0.001	3.87 (3.42–4.37)	<0.001
Relative weight (per unit)	0.85 (0.78–0.92)	0.091	1.15 (1.09–1.22)	<0.001

DRG: Diagnosis-Related Group, HR: Hazard Ratios.

## Data Availability

The data that support the findings of this study are available from the corresponding author upon reasonable request.

## Supplementary Materials

The following supporting information can be downloaded at: https://www.mdpi.com/article/10.3390/jcm14217717/s1. Table S1: Baseline characteristics of older adults hospitalized with hip fracture, stratified by hospital outcome (Survived/Deceased); Table S2: Baseline characteristics of older adults hospitalized with hip fracture, stratified by surgical treatment (Yes/No).

## Abbreviations

DRG	Diagnosis-Related Group
FONASA	National Health Fund (Fondo Nacional de Salud, Chile)
ICD-10	International Classification of Diseases
