# Supplementary material for: In-Hospital Mortality Among 40,253 Older Adults with Hip Fracture: Survival Outcomes and Multivariate Analysis in a Chilean Cohort
Source: J Clin Med. 2025 Oct 30;14(21):7717. doi: 10.3390/jcm14217717 (PMC12608696; doi:10.3390/jcm14217717)
Supplement: Supplementary file 1 [file jcm-14-07717-s001.zip › jcm-3934000-supplementary.pdf]

Table S1. Baseline characteristics of older adults hospitalized with hip fracture, stratified by hospital outcome (Survived/Deceased).

**Variable**	**Survived (n = 38,840; 96.49%)**	**Deceased (n = 1413; 3.51%)**
Age, years	81.79 ± 9.07	86.08 ± 8.35
Female sex, n (%)	29,908 (77.00%)	1000 (70.77%)
>=3 comorbidities, n (%)	34,656 (89.23%)	1382 (97.81%)
DRG severity: high, n (%)	6937 (17.86%)	1101 (77.92%)
Relative weight	1.45 ± 0.50	1.57 ± 1.18
Days of hospitalization	12.60 ± 13.47	16.54 ± 23.06
Surgical treatment, n (%)	34,807 (89.62%)	633 (44.80%)

Table S2. Baseline characteristics of older adults hospitalized with hip fracture, stratified by surgical treatment (Yes/No).

**Variable**	**Surgery (n = 35,440; 88.04%)**	**No Surgery (n = 4813; 11.96%)**
Age, years	81.74 ± 9.03	83.38 ± 9.32
Female sex, n (%)	27,328 (77.11%)	3580 (74.38%)
≥3 comorbidities, n (%)	31,642 (89.28%)	4396 (91.34%)
DRG severity: high, n (%)	6639 (18.73%)	1399 (29.07%)
Relative weight	1.56 ± 0.48	0.74 ± 0.38
Days of hospitalization	13.01 ± 13.80	10.68 ± 14.74
In-hospital mortality, n (%)	633 (1.79%)	780 (16.21%)
